# Factors related to the increasing number of seriously injured cyclists and pedestrians in a Swedish urban region 2003–17

**DOI:** 10.1093/pubmed/fdz064

**Published:** 2019-06-18

**Authors:** Astrid Värnild, Per Tillgren, Peter Larm

**Affiliations:** 1 School of Health, Care and Social Welfare, Mälardalen University, Sweden; 2 Department of Clinical Neuroscience, Karolinska Institutet, Sweden

**Keywords:** accidents, epidemiology, management and policy

## Abstract

**Background:**

The number of seriously injured unprotected road users has increased during implementation of a road safety policy Vision Zero. The aim of the study is to identify factors associated with the increase in serious injuries among cyclists and pedestrians (even single pedestrian accidents) that occurred in an urban road space in a Swedish region 2003–17. The urban road space includes roads, pavements and tracks for walking and cycling.

**Methods:**

Data were retrieved from STRADA (Swedish Traffic Accident Data Acquisition) and NVDB (National Road Database). Descriptive statistics and logistic regression with odds ratios for sex, age and part of road space were assessed.

**Results:**

The number of seriously injured cyclists and pedestrians more than doubled from 2003 to 2017, with the greatest increase for pedestrians. Older age increased the probability of serious injury since 2012 for the group ≥ 80 years and since 2015 for the group 65–79 years. No significant effect of sex. Most injuries occur in areas not transformed by Vision Zero.

**Conclusions:**

An increasing number of elderly persons in the generation born in the 1940s and increased life expectancy are important factors. There is a need to increase road safety measures that also promote active mobility.

## Background

WHO reports that 20–50 million people are injured in road traffic every year, many of whom are left with permanent disabilities^1^. Fatalities have decreased in many high-income countries since the turn of the millennium^2^. For example in the EU, which has the safest roads globally, fatalities have decreased by 20% since 2010^3^. Nevertheless, road traffic crashes are the eighth leading cause of death globally^1^. Thus, Agenda 2030 has the goal of halving the number of road injuries between 2010 and 2020, and providing access to safe cities by 2030. This will be done by improving road safety, with special attention being given to those in vulnerable situations^4^. Different approaches to road safety management have been developed. Although, there may be differences between countries in terms of what actions are taken and how they are realized and prioritized, some common principles can be seen such as appropriate speed limits for roads, mandatory seat belt and helmet use, and efforts to curb alcohol and drug use in traffic^1,2^. Many countries have made advances in road safety management by setting quantitative targets and adopting programmes for implementing measures to reach the targets^5^. Vision Zero is a well-known Swedish policy^1,6–8^ with the goal of reducing fatalities and serious injuries in road traffic to zero^9,10^. Rural roads have been redesigned with median barriers or provided with speed-cameras in conjunction with a national speed-limit revision. On urban roads, municipalities have had the possibility to reduce speeds to 30 km/h with the help of traffic bumps or in roundabouts, and to reduce speed limits from 50 km/h to 30 or 40 km/h more generally^11–13^. During the implementation of Vision Zero, the number of fatalities has decreased in Sweden^1,14^, but whether this trend also holds for seriously injured road users is not confirmed^15–17^.

A previous unpublished study shows that, although the number of seriously injured car occupants on national roads decreased when Vision Zero was implemented, the number of seriously injured unprotected road users increased^18^. This finding indicates that the decrease in seriously injured car drivers on national roads is countered by an increased number of seriously injured unprotected road users, which may have serious implications for the Swedish road safety management system. Further, single pedestrian crashes are not included in the target of Vision Zero because they are not usually defined as road traffic crashes^17,18^. Still, single pedestrian crashes occur in areas also used by other road users, especially cyclists. Whether or not single pedestrian crashes should be included in the definition of road traffic crashes has been discussed^19–21^. Research on the implications of road safety management systems for unprotected road users is scarce^22^, probably because single pedestrian crashes often have an unclearly articulated position in studies, and pedestrian crashes are often presumed only to refer to collisions with other road users^23^.

However, given the concurrent increase of obesity and obesity-related morbidity^24^, the Swedish government has emphasized the importance of developing infrastructure that stimulates more walking and cycling, which is in line with Agenda 2030^25^. Much has been done in Sweden to reach road safety targets during the implementation of Vision Zero^1,14^. Despite this, the number of seriously injured unprotected road users has increased, most of whom are cyclists and pedestrians^16^ in urban road spaces, which besides roads also include pavements and tracks for walking and cycling^22,26^. More than 80% of all seriously injured road users in an urban road space are cyclists and pedestrians, almost evenly distributed between cyclists and pedestrians^16^. Importantly, the reason why the number of seriously injured unprotected road users has increased is unknown. In Sweden, the municipalities are the responsible road authorities for urban areas, and thus are responsible for the design of infrastructure and for most road space maintenance. It is therefore important to adopt a regional perspective when trying to understand the increased number of seriously injured cyclists and pedestrians in urban areas.

The present study aims to identify factors associated with serious injuries of cyclists and pedestrians including also single pedestrian accidents in an urban road space during the implementation of Vision Zero between 2003 and 2017 in the Swedish Region of Västmanland. Three objectives are addressed: (a) How has the number of serious injuries among cyclists and pedestrians developed in the region from 2003 to 2017? (b) What role do demographic factors including sex and age play in this development? (c) What role do different parts of the road space linked to Vision Zero measures play in this development?

## Methods

### Data about seriously injured cyclists and pedestrians

Data was retrieved from the Swedish registry STRADA (Swedish Traffic Accident Data Acquisition)^27^. The data in STRADA has been collected since the year 2000 by emergency hospitals for patients, regardless of whether they were hospitalized or sent home. Since 2003, this data has been complemented by data from police reports. In Sweden, only two regions have submitted data from the start, and Region Västmanland is one of these. The registry has been built up successively, and in 2016, STRADA became a national registry with data from all regions^28^.

For this study, all cyclists and pedestrians seriously injured (also from single pedestrian accidents) in an urban road space consisting of roads, pavements and tracks in Region Västmanland during the period 2003–17 were identified. This yielded a total of 403 cases, of which 197 concern cyclists and 206 pedestrians. The geographic location for each injury included in the study was also examined in the National Road Database (NVDB)^29^ to determine if the location had been transformed by Vision Zero when the injury occurred. This study used cross sectional data for the region that was annually collected for a period of fifteen years.

### Measurements

#### Serious injuries

Health care data in STRADA is linked to the AIS-scale (Abbreviated Injury Scale). The scale estimates the severity of every injury on a six-degree scale where AIS 1 is a minor injury and AIS 6 is a maximum-severity injury related to mortality, but the scale does not estimate an injury’s long-term consequences^30^. Therefore, the ISS-score (Injury Severity Score) is used, which is an adaptation of the AIS-scale where the AIS scores for a person’s most serious injuries from at most three of six defined bodily regions are squared and summed. The ISS value varies between 1 and 75, and a seriously injured person is defined by an ISS score >8, according to the STRADA registry’s definition; this cut off is used in the present study to define serious injury.

#### The road space

The road space was divided into non-road areas, such as pavements and tracks for cycling and walking, and Vision Zero and Non-Vision Zero roads respectively. Vision Zero has primarily focused on safe design of roads where cyclists and pedestrians encounter motor vehicles. Therefore, only roads are divided into Non-Vision Zero and Vision Zero road areas. Vision Zero roads are characterized by safe road/pedestrian crossing (reduced speed limit of 30 km/h and over- or underpass), reduced speed limit (40 km/h), and roundabouts. Of 403 injured cyclists and pedestrians, 68 were injured on Vision Zero roads and 95 on Non-Vision Zero roads with a speed limit of 50 km/h. Further, 240 cyclists and pedestrians were injured on tracks and pavements without speed limits. Of these, 130 were injured on tracks for cycling and walking, and 110 on pavements.

#### Cause of crash related to maintenance or other causes

This concerns serious injuries related to maintenance, defined as seasonal characteristics of the road space such as ice, snow, gravel and wet leaves, but potholes and loose paving stones are also included as confounders. Other causes are design problems of the road space like obstacles, curbs and other edges, and problems related to the bicycle’s functioning or mistakes by the individual cyclist or pedestrian, like getting a bag stuck in the bicycle wheel or having bad shoes.

#### Municipality size

Region Västmanland is a Swedish metropolitan area, and the proportion of the population that lives in an urban area has increased from 86–88% between 2005 and 201531–32. The region consists of 10 municipalities, with one municipality having 55% of the inhabitants (271,000). None of the other nine municipalities in the region has more than 26 000 inhabitants^33^. Whether the injury occurred in an urban municipality (≥ 100,000 inhabitants) is included as a confounder.

### Statistical analysis

In order to describe the development of the incidence of serious injuries among cyclists and pedestrians in the region from 2003 to 2017, the number of seriously injured cyclists and pedestrians in five three-year intervals was initially identified and presented. Three-year intervals were used to reduce the effect of large fluctuations that can occur between single years. Secondly, demographics and injury characteristics for seriously injured persons in the region were presented with mean values for age and percentages for all other characteristics. Thirdly, the important demographic characteristics, including age and sex, for the development of the incidence of serious injuries from 2003 to 2017 were analysed with a series of multiple logistic regression analyses, yielding Odds Ratios (OR) and 95% Confidence Intervals (CI). Odds ratios for sex and age in four three-year periods, compared to the reference period 2003–05, were calculated. The analyses were adjusted for cause of crash and municipality size. Finally, the influence of Vision Zero roads was studied using a similar series of multiple logistic regression analyses related to the five given time periods using Non-Vision Zero roads as a reference.

### Ethics

This study has been approved by the Swedish Ethical Board in Uppsala, Sweden (Dnr 2015/016/1)

## Results

**Fig. 1:**
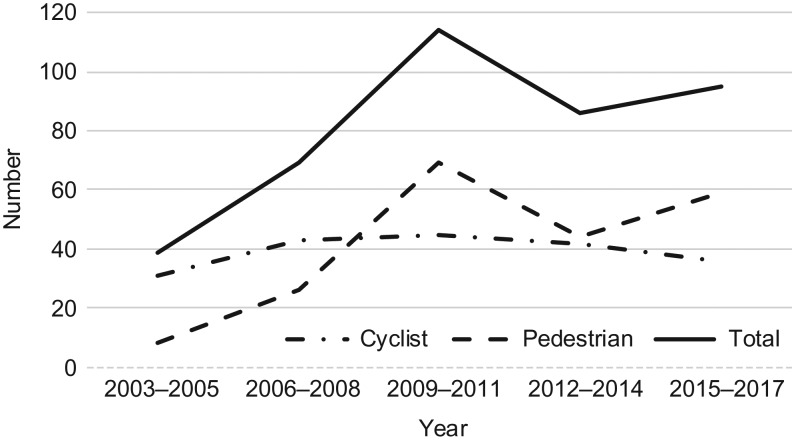
Number of seriously injured cyclists and pedestrians.

The number of seriously injured cyclists and pedestrians in urban areas of Region Västmanland more than doubled from 2003 to 2017 and almost tripled from 2003–05 to 2009–11, only to decrease thereafter, Figure 1. When disentangling cyclists from pedestrians, we found that the increase was attributable to pedestrians with a more than sevenfold increase, while the number of seriously injured cyclists initially increased but then stood still. There were fewer seriously injured pedestrians than cyclists from the start, but beginning in 2009–11 there were more seriously injured pedestrians than cyclists.

Further, demographic characteristics of seriously injured cyclists and pedestrians, and crash circumstances, are described in Table 1. Mean age increased during the study period from 53.2 years to 64.5 years for cyclists and from 60.4 years to 68.8 years for pedestrians. The proportion of cyclists and pedestrians that were seriously injured on Vision Zero roads increased from 7.7% to 26.3%, while the proportion that were seriously injured on Non-Vision Zero roads decreased from 41% to 21.1%, although the relative numbers were stable. For all the periods, more than 50% of the injuries occurred on pavements and tracks.

**Table 1:** Demographics and injury characteristics of serious injuries

Variables	2003–2005	2006–2008	2009–2011	2012–2014	2015–2017
Road user % (n)					
Cyclists *n* = 197	79.5% (31)	62.3% (43)	39.5% (45)	48.8% (42)	37.9% (36)
Pedestrians *n* = 206	20.5% (8)	37.7% (26)	60.5% (69)	51.2% (44)	62.1% (59)
Sex % (n)					
Men *n* = 207	48.7% (19)	49.3% (34)	56.1% (64)	45.3% (39)	53.7% (51)
Women *n* = 196	51.3% (20)	50.7% (35)	43.9% (50)	54.7% (47)	46.3% (44)
Mean age (Stand. Dev.)					
Cyclists *n* = 197	53.2 (24.7)	57.5 (23.2)	53.5 (20.4)	59.6 (20.7)	64.5 (19.3)
Pedestrians *n* = 206	60.4 (20.5)	61.4 (23.5)	66.9 (20.5)	69.6 (18.8)	68.8 (19.6)
Road space % (n)					
Non-Vision Zero road *n* = 95	41.0% (16)	29.0% (20)	14.9% (17)	25.6% (22)	21.1% (20)
Vision Zero road *n* = 68	7.7% (3)	13.0% (9)	11.4% (13)	20.9% (18)	26.3% (25)
Pavement/track *n* = 240	51.3% (20)	58.0% (40)	73.7% (84)	53.5% (46)	52.6% (50)
Cause of crash rela. to… % (n)					
Maintenance *n* = 175	43.6% (17)	23.2% (16)	46.5% (53)	43.0% (37)	54.7% (52)
Other causes *n* = 228	56.4% (22)	76.8% (53)	53.5% (61)	57.0%(49)	45.3% (43)
Municipality-urban area % (n)					
≥100,000 inhabitants *n* = 253	69.2% (27)	59.4% (41)	67.5% (77)	57.0% (49)	62.1% (59)
<100,000 inhabitants *n* = 150	30.8% (12)	40.6% (28)	32.5% (37)	43.0% (37)	37.9% (36)

The logistic regression analysis of demographic factors including age and sex showed that sex had no influence during the period, Table 2. In contrast, older age increased the probability of serious injuries among cyclists and pedestrians from 2012 and forward. In particular, the age group 65–79 years was 6.09 (CI 1.86–19.89) times more likely to be seriously injured than the youngest age category during 2015–17. Similarly, compared to the youngest age category, the oldest age group (≥80 years) was associated with increased probability of serious injuries (OR 4.33, CI 1.18–15.89) during 2012–14, and this increased during 2015–17 (OR 9.81, CI 2.33–41.26). All multiple logistic regression analyses were adjusted for cause of crash and municipality size. The importance of older age remained when road space was included in the analysis, Table 3.

**Table 2:** Adjusted odds ratios and 95% confidence intervals for associations between demographic characteristics and serious injuries stratified by three-year intervals

Variables	2006–2008	2009–2011	2012–2014	2015–2017
	OR (C.I.)	OR (C.I.)	OR (C.I.)	OR (C.I.)
Sex				
Men (reference)	1.00	1.00	1.00	1.00
Women	1.07 (0.46–2.48)	0.69 (0.32–1.48)	1.18 (0.52–2.65)	0.46 (0.19–1.13)
Age				
0–49 (reference)	1.00	1.00	1.00	1.00
50–64	1.18 (0.38–3.64)	1.35 (0.48–3.81)	1.09 (0.34–3.52)	2.95 (0.88–9.85)
65–79	0.87 (0.31–2.49)	1.31 (0.51–3.35)	2.10 (0.78–5.63)	6.09 (1.86–19.89)
≥ 80	2.99 (0.80–11.24)	2.99 (0.84–10.65)	4.33 (1.18–15.89)	9.81 (2.33–41.26)

Note: Adjusted for cause of crash and municipality size.

**Table 3:** Adjusted odds ratios and 95% confidence intervals for the association between road space characteristics and serious injuries stratified by three-year intervals

Variables	2006–2008	2009–2011	2012–2014	2015–2017
	OR (C.I.)	OR (C.I.)	OR (C.I.)	OR (C.I.)
Sex				
Men (reference)	1.00	1.00	1.00	1.00
Women	1.04 (0.44–2.45)	0.84 (0.38–1.87)	1.19 (0.52–2.71)	0.47 (0.19–1.17)
Age				
0–49 (reference)	1.00	1.00	1.00	1.00
50–64	1.04 (0.31–3.45)	1.71 (0.57–5.16)	1.07 (0.31–3.64)	3.18 (0.91–11.13)
65–79	0.92 (0.32–2.66)	1.44 (0.54–3.83)	2.05 (0.75–5.59)	6.42 (1.89–21.88)
≥ 80	2.86 (0.76–10.78)	2.88 (0.78–10.69)	4.00 (1.06–15.06)	7.32 (1.61–33.34)
Road space				
N-VZ road (reference)	1.00	1.00	1.00	1.00
Vision-Zero road	2.84 (0.57–14.10)	4.15 (0.95–18.03)	4.36 (1.04–18.31)	5.91 (1.36–25.65)
Pavement/Track	2.17 (0.83–5.66)	4.13 (1.66–10.29)	1.75 (0.60–5.11)	2.11 (0.83–5.37)

Note: Adjusted for cause of crash and municipality size.

Logistic regression analyses in Table 3 also showed that roads having been transformed by Vision Zero increased the probability of serious injuries compared to Non-Vision Zero roads during the time periods 2012–14 (OR 4.36, CI 1.04–18.31) and 2015–17 (OR 5.91, CI 1.36–25.65). Cyclists and pedestrians also had an increased probability of serious injuries on pavements and tracks compared to Non-Vision Zero roads during 2009–11 (OR 4.13, CI 1.66–10.29).

## Discussion

### Main findings of this study

The number of seriously injured cyclists and pedestrians more than doubled during the period when Vision Zero was being implemented, most of which increase can be attributed to pedestrians while the number of seriously injured cyclists stood still. One reason for the increased number of seriously injured pedestrians may be the increase in population during the study period^33^. On the other hand, in a previous unpublished study we showed that the increase of serious injuries among unprotected road users was independent of changes in population 2003–14^18^. Another explanation may be that, nationally, the distance travelled by pedestrians increased by 50% between 1995 and 2014, while distance travelled by cyclists decreased somewhat^34^. Thus, the increase in seriously injured pedestrians may due to their being more physically active. Importantly, however, our finding shows that when the Vision Zero programme-which predominately focused on reducing fatalities on the roads among car users and cars’ collisions with unprotected road users^9,10^ – was implemented in Sweden, the number of seriously injured unprotected road users actually increased instead of decreasing.

From 2003 to 2017, the mean age of seriously injured pedestrians and cyclists increased for cyclists from 53.2 to 64.5 years, and for pedestrians from 60.4 to 68.8 years of age. The multivariate analyses showed an increased probability of serious injuries from 2012 for the ≥80 years of age group, together with an increased probability for the 65–79 years of age group from 2015. One possible explanation for the increased number of serious injuries among older cyclists and pedestrians in the Region of Västmanland is that people are getting older in Europe as a result of the 1940s baby-boom generation and an increased life expectancy^21,35,36^. In Region Västmanland, the populations of 65–79 year-olds and ≥80 year-olds increased during 2015–17 in comparison with 2003–05, while the population of persons younger than 50 years was unchanged, and the population of 50–64 year-olds decreased^33^. Thus, the increasing age of seriously injured cyclists and pedestrians may merely reflect the increasing age of the population. Older unprotected road users are also a vulnerable group in the road traffic. A national study about pedestrians covering 2003–08 reports 83.9% more injuries for the ≥75 years of age group than for the 65–74 years of age group^37^. Nationally, the 80 years and older group will increase by 50% between 2018 and 2028^38^. It is therefore important that measures are taken to protect older unprotected road users.

The transformation of roads from Non-Vision Zero to Vision Zero roads also had a direct effect on seriously injured cyclists and pedestrians in our study. Results from the multivariate analyses showed an increased probability of being seriously injured on Vision Zero roads compared to Non-Vision Zero roads, from 2012. Since the start, the focus of Vision Zero has been to design safe roads and to prevent collisions between cars and unprotected road users by means of speed reductions on urban roads to 30 km/h^9,10^. However, two-thirds of all road traffic casualties in Europe are unprotected road users, and two-thirds of these are injured in single crashes because of ageing population and urbanization^21^. It thus appears that unprotected road users involved in single crashes should be included in the measures undertaken within Vision Zero.

### What is already known on this topic

To our knowledge, no previous study has addressed the development of seriously injured cyclists and pedestrians in urban traffic during a period when the road safety measure of Vision Zero was implemented. Previous studies on road safety have focused on injuries from multiple crashes and single crashes for cyclists. Pedestrians in single crashes have been excluded from these studies^19,20,22,39^.

### What this study adds

This study has multiple implications for public health, given its finding that seriously injured older unprotected road users in urban areas increased. First, the aging population and increasing life expectancy will require traffic management systems that prevent fatalities and serious injuries among unprotected road users. Two, this is particularly important because Agenda 2030 has the goal of halving the number of road injuries and providing access to safe cities. Three, given that physical activities such as walking and cycling have large health benefits, designing a road management system that encourages physical activity should be of high priority. The study also adds a regional perspective, which is important since in Sweden, municipalities are the responsible road authorities for implementing Vision Zero urban areas. The region of Västmanland was one of two regions that reported data to STRADA from 2003 and forward enabling us to identify serious injuries during a long time period. All data in the study are linked to a road space defined by demarcating a particular subset of public space^22,26^. Defining the road space is important in order to clarify the environment of the examined area in a study.

### Limitations of the study

Although the study includes data covering fifteen years, it includes relatively few injured cyclists and pedestrians, giving wide confidence intervals that impair statistical power. A larger population may have yielded more significant associations. The study also includes a limited number of demographic factors, which limits the interpretation of what role demographic factors have for the increased number of serious injuries.

## Conclusion

The number of seriously injured cyclists and pedestrians has increased in a regional urban area during the implementation of Vision Zero during 2003–17, with pedestrians being especially affected. There is therefore a great need to increase road safety measures for cyclists and pedestrians, and this would also promote public health by improving the conditions for more active mobility.

The probability of serious injuries among cyclists and pedestrians has been increasing since 2012 for the 80 years and older group, and since 2015 for the 65 years and older group. The increasing number of old people in the 1940s baby-boom generation together with increased life expectancy are an incipient public health problem.
